# Cardiovascular Autonomic Neuropathy Is an Independent Risk Factor for Left Ventricular Diastolic Dysfunction in Patients with Type 2 Diabetes

**DOI:** 10.1155/2017/3270617

**Published:** 2017-05-30

**Authors:** Jiewen Jin, Weimin Wang, Liangying Zhu, Tianwei Gu, Qing Niu, Ping Li, Yan Bi, Dalong Zhu

**Affiliations:** ^1^Department of Endocrinology, Drum Tower Hospital Affiliated to Nanjing University Medical School, Nanjing, Jiangsu, China; ^2^Department of Endocrinology, Drum Tower Clinical Medical College, Nanjing Medical University, Jiangsu, China

## Abstract

**Aim:**

This study aimed to evaluate the association between cardiovascular autonomic neuropathy (CAN) and left ventricular diastolic dysfunction (LVDD) in type 2 diabetes patients.

**Methods:**

315 type 2 diabetes patients from inpatients of Drum Tower Hospital were included and classified into no CAN (NCAN), possible CAN (PCAN), and definite CAN (DCAN) based on cardiovascular autonomic reflex tests. The left ventricular diastolic function was assessed by tissue Doppler imaging echocardiography.

**Results:**

The distribution of NCAN, PCAN, and DCAN was 11.4%, 51.1%, and 37.5%, respectively. The proportion of LVDD increased among the groups of NCAN, PCAN, and DCAN (39.4%, 45.3%, and 68.0%, *P* = 0.001). Patients with DCAN had higher filling pressure (*E*/*e*′ ratio) (10.9 ± 2.7 versus 9.4 ± 2.8, *P* = 0.013) and impaired diastolic performance (*e*′) (6.8 ± 1.7 versus 8.6 ± 2.4, *P* = 0.004) compared with NCAN. CAN was found to be an independent risk factor for LVDD from the multivariate regression analysis (OR = 1.628, *P* = 0.009, 95% CI 1.131–2.344).

**Conclusions:**

Our results indicated that CAN was an independent risk marker for the presence of LVDD in patients with diabetes. Early diagnosis and treatment of CAN are advocated for preventing LVDD in type 2 diabetes.

## 1. Introduction

Cardiovascular autonomic neuropathy (CAN) is one of the most common complications in patients with diabetes. It is defined as an impairment of cardiac autonomic nerve fibers in the cardiovascular system after excluding other causes [1]. Decreased heart rate variation (HRV) during tilting up, deep breathing, and Valsalva's maneuver are early manifestations of CAN [2]. The prevalence of CAN varies according to different diagnostic criteria [3] and ranges between 16.1% and 22.1% on performing at least two abnormal cardiovascular heart rate tests [1, 4]. CAN prevalence increases with age (up to 44% in patients with type 2 diabetes aged over 65 years) and duration of diabetes (up to 65% in long-standing type 2 diabetes) [1, 5].

It was demonstrated that CAN is associated with increased risk of morbidity and mortality in patients with diabetes [6, 7]. After correcting for age, duration, glucose control, and cardiovascular risk factors, CAN was shown to be an independent predictor of cardiovascular morbidity and mortality [7–9]. Several mechanisms could explain how CAN promotes mortality, including silent myocardial ischemia, fatal arrhythmia due to QT prolongation, and impaired respiratory response to hypoxia [10, 11]. However, the exact mechanism remains unclear.

Patients with diabetes show a high frequency of heart failure accompanied by an increased risk of mortality [12]. Diastolic heart failure is a common form of heart failure with preserved ejection fraction and characterized by left ventricular diastolic dysfunction (LVDD) [13]. LVDD manifests as left ventricular filling impairment, relaxation delay, and increased stiffness [14]. Previous studies have revealed associations of CAN with altered left ventricular relaxation and filling, increased left ventricular mass, left ventricular hypertrophy, and impairment of myocardial blood flow regulation [15, 16].

In the present study, we aimed to clarify the relationship between CAN and LVDD in Chinese patients with type 2 diabetes.

## 2. Methods

### 2.1. Subjects

This study included 315 patients with type 2 diabetes admitted to Drum Tower Hospital, Nanjing, China, from September 2015 to December 2016. Inclusion criteria were confirmed type 2 diabetes according to American Diabetes Association criteria and age between 18 and 75 years. Patients with stroke, hypothyroidism, alcohol addiction, and other causes of neuropathy except diabetes were excluded. This study was approved by the ethics committee of Drum Tower Hospital.

### 2.2. CAN Assessment

CAN was assessed according to four cardiovascular autonomic reflex tests (CARTs): heart rate variation during deep breathing (HRV_DP), HRV during Valsalva maneuver (HRV_Valsalva), HRV during the lying-to-standing test (HRV 30 : 15), and blood pressure variability (BPV) in response to standing up.

In HRV_DP, the patients were instructed to rest in the supine position at least 5 min before the test and breathe deeply at a rate of six breaths per minute while being monitored on a 12-lead electrocardiography (25 mm/s). The maximum and minimum heart rates during each breathing cycle were measured, and the mean difference of the six cycles was calculated [17].

In HRV_Valsalva, the patients were instructed to perform forced exhalation with a pressure of 40 mmHg for 15 s. The ratio of the maximum R-R interval after the maneuver to the minimum R-R interval during the maneuver was calculated. The test was performed three times, and the mean ratio was used [17].

In HRV 30 : 15, the patients were instructed to rest in the supine position at least 5 min before the test. The 30 : 15 ratio was determined as the longest R-R interval (measured around the 30th beat after standing up) divided by the shortest R-R interval (measured around the 15th beat after standing up) [17].

For BPV in response to standing up, the patients were instructed to rest in the supine position at least 5 min before the test. Systolic pressure was measured when changing from the supine to the upright position, and measurements were taken every minute for at least 3 min [17].

Each test had normative values according to previous report [18]. Two or more abnormalities in CARTs were defined as definite CAN (DCAN), one abnormality in CARTs was defined as possible CAN (PCAN), and no abnormality in CARTs was defined as no CAN (NCAN).

### 2.3. Echocardiography

Patients underwent echocardiography examination using the Philips 5500 ultrasound machine instrument, with data analyzed with the corollary software (Philips, WA, USA). Both two-dimensional and tissue Doppler imaging (TDI) were performed. Left ventricular ejection fraction (using the modified Simpson's biplane method), traditional Doppler parameters of peak velocities of early (*E*) and atrial (*A*) transmitral flow velocities, and TDI parameter of peak early diastolic (*e*′) velocities were evaluated. The TDI parameter was obtained by placing a tissue Doppler volume at the septal annulus in the apical four-chamber view. *E*/*e*′ ratio and *e*′ were calculated as measures of left ventricular filling pressure and diastolic performance, respectively [19]. Left ventricular mass index (LVMI) was defined as the anatomic mass divided by the body surface area of the patient. *E*/*e*′ ratio, *E*/*A* ratio, and *e*′ velocity were used to diagnose LVDD according to the European Association of Echocardiography consensus paper [20].

### 2.4. Statistics

Data were presented as mean ± standard deviation (SD) for continuous variables and percentages for dichotomous variables. SPSS22.0 (SPSS, IL, USA) was used for all statistical analyses. Student's *t* test was used to assess continuous variables, and chi-square test or Fisher exact test was employed for dichotomous variables. Bivariate correlation analyses were performed to identify the associations of CAN assessment parameters with echocardiography indicators for diastolic dysfunction. A multivariable logistic regression model was applied, with the LVDD status as the dependent variable, and age, gender, diabetic duration, smoking, low density lipoprotein (LDL) cholesterol, hemoglobin A1c (HbA1c), pulse pressure, corrected QT interval (QTc), and CAN as independent variables to explore whether CAN is an independent risk factor for LVDD.

## 3. Results

### 3.1. Patient Characteristics

Among the 315 patients with type 2 diabetes, 11.4% had NCAN, 51.1% had PCAN, and 37.5% had DCAN. Among the three groups, patients with DCAN were older (60.4 ± 8.0 y in DCAN, 57.1 ± 11.2 y in PCAN, and 54.2 ± 11.8 y in NCAN, *P* = 0.002), had longer diabetic duration (12.9 ± 7.6 y, 10.2 ± 7.4 y, and 7.9 ± 6.3 y, *P* < 0.001) with a higher low density lipoprotein (LDL) cholesterol level (2.52 ± 0.82 mmol/L, 2.24 ± 0.84 mmol/L, and 2.28 ± 0.63 mmol/L, *P* = 0.022), and had higher proportion of family history of diabetes (52.5%, 36.8%, and 22.2%, *P* = 0.002) compared with the PCAN and NCAN groups. Pulse pressure and corrected QT intervals (QTc) were significantly different among the three groups. 48.1% of the patients with DCAN had diabetic retinopathy and 52.3% had diabetic sensorimotor polyneuropathy, indicating much higher rates compared with the other two groups (Table 1).

### 3.2. Left Ventricular Diastolic Function and LVDD Proportion

All patients had normal left ventricular ejection fraction. Conventional echocardiography measures of left ventricular diastolic function showed higher *A* values (0.88 ± 0.16 cm/s in DCAN, 0.77 ± 0.20 cm/s in PCAN, and 0.78 ± 0.20 cm/s in NCAN, *P* < 0.001) and lower *E*/*A* ratios (0.83 ± 0.23, 0.99 ± 0.32, and 1.01 ± 0.36, *P* < 0.001) in patients with DCAN compared with those with PCAN and NCAN. Left ventricular (LV) mass and LVMI showed significant differences among the subjects with NCAN, PCAN, and DCAN (LV mass, 145.9 ± 28.6 g, 153.0 ± 31.6 g, and 167.5 ± 40.3 g, *P* < 0.001; LVMI, 83.8 ± 13.3 g/m^2^, 87.1 ± 16.1 g/m^2^, and 95.8 ± 19.1 g/m^2^, *P* < 0.001). TDI measures indicated higher filling pressure (*E*/*e*′ ratio) (10.9 ± 2.7 in DCAN, 9.8 ± 2.8 in PCAN, and 9.4 ± 2.8 in NCAN, *P* = 0.006) and impaired diastolic performance (*e*′) (6.8 ± 1.7 cm/s, 8.3 ± 3.1 cm/s, and 8.6 ± 2.4 cm/s, *P* < 0.001) in DCAN patients compared with the PCAN and NCAN groups (Table 2). LVDD was found in 45.1% of all studied type 2 diabetes patients and increased in the NCAN (39.4%), PCAN (45.3%), and DCAN (68.0%) groups (*P* = 0.001) (Table 2).

### 3.3. Associations of LVDD, Pulse Pressure, and QTc with CAN

LVDD, pulse pressure, and QTc are risk factors for mortality. The *e*′ value was correlated with HRV 30 : 15 (*r* = 0.224, *P* < 0.001), HRV_DP (*r* = 0.227, *P* = 0.001), and HRV_Valsalva (*r* = 0.216, *P* = 0.031). Other indicators of diastolic dysfunction, including *E*/*e*′ ratio, *E*/*A* ratio, LV mass, and LVMI, showed significant associations with CAN parameters (Table 3). Pulse pressure was correlated with HRV_DP (*r* = −0.197, *P* = 0.001) and HRV 30 : 15 (*r* = −0.209, *P* < 0.001), and QTc was correlated with HRV 30 : 15 (*r* = −0.161, *P* = 0.005) and BPV (*r* = −0.153, *P* = 0.008). Pulse pressure was also correlated with QTc (*r* = 0.301, *P* < 0.001). CAN stage was shown to be an independent risk factor for LVDD (OR = 1.628, *P* = 0.009, 95% CI 1.131–2.344) after adjustment for known determinants of LVDD, including age, gender, diabetic duration, smoking, LDL, HbA1c, pulse pressure, and QTc (Table 4).

## 4. Discussion

This study demonstrated a close correlation between CAN and LVDD in type 2 diabetes. The severity of left ventricular filling pressure and diastolic performance dysfunction increased among the NCAN, PCAN and DCAN groups. CAN was found to be an independent risk factor for LVDD occurrence after multivariate adjustment. Among the four CAN parameters, HRV 30 : 15, HRV_DP, and HRV_Valsalva, which reflect the parasympathetic function, were associated with LVDD.

CAN represents an impairment of autonomic nerve fibers that could result in abnormalities in heart rate variation and vascular dynamics [10]. It is usually assessed and quantified by CARTs [1]. Patients with diabetes were classified as NCAN, PCAN, and DCAN in the present study, according to the above-mentioned method. Meanwhile, CAN was also revealed to be an independent predictor of cardiac events and death after multivariate analysis, and the maldistribution of sympathetic innervation might be involved [6]. However, how CAN promotes cardiovascular morbidity and mortality remains unclear. LVDD, increased pulse pressure, and prolonged QT interval are all independent risk factors for high mortality, which might explain the aforementioned phenomenon.

LVDD is characterized by an impairment of left ventricular passive filling and relaxation, and considered the earliest manifestation of diabetic cardiomyopathy [15], which is associated with the development of heart failure and predictive of all-cause mortality [21]. The prevalence of LVDD in the general population is approximately 20% to 30%, and increases with age and comorbidities, such as diabetes and hypertension [21]. In patients with type 2 diabetes, LVDD prevalence is 40% to 75% as detected by echocardiography [22], in agreement with the current findings (45.1% LVDD in all patients enrolled). An association of CAN with LVDD is well documented [15, 23, 24]. In type 1 diabetes, CAN (abnormal R-R variation) is correlated with higher left ventricular mass and cardiac output, as assessed by cardiac magnetic resonance imaging in the Diabetes Control and Complications Trial/Epidemiology of Diabetes Interventions and Complications cohort [15]. CAN was further proved to be independently correlated with subclinical left ventricular dysfunction in type 1 diabetic patients with normal albuminuria, including systolic and diastolic functions, and coronary artery calcium score, evaluated by computed tomography [3]. Among the patients who underwent coronary angiography, type 2 diabetics with CAN showed reduced *e*′ and increased *E*/*e*′ ratio as assessed by TDI, after adjustment for age, sex, coronary artery disease, hypertension, and HbA1c [25]. Sacre et al. revealed that CAN markers have independent associations with resting *e*′, and diastolic impairment central to autonomic cardiopathy was linked to cardiac sympathetic denervation, which was carefully assessed by myocardial uptake of ^123^I-MIBG [16]. The present study confirmed the close relationship between CAN and LVDD in type 2 diabetes and highlighted the important role of parasympathetic dysfunction, especially the 30 : 15 test, in cases with LVDD. The previous studies mainly focused on the presence of CAN and LVDD, with CAN severity often overlooked. Here, we further divided the patients into three groups according to abnormality number in CARTs. With the number of abnormalities increased, LVDD proportion also increased as well as indicators of diastolic dysfunction. These findings suggested that the more severe the CAN, the higher the LVDD occurrence and the more impaired diastolic function; therefore, early detection and intervention of CAN may help prevent LVDD.

QT interval prolongation was shown to be correlated with CAN in type 1 diabetes [26, 27]. In the current study, increased QTc was associated with CAN in type 2 diabetes; further analysis showed inverse associations of QTs with HRV 30 : 15 and BPV. There is a strong evidence in both type 1 and type 2 diabetes patients that prolonged QT interval might constitute a predictor of cardiac and all-cause mortality [28]. We hypothesized that QT prolongation was one of the mechanisms by which CAN promotes mortality.

Pulse pressure is positively correlated with cardiovascular mortality in diabetes [29]. In addition, increased pulse pressure is accompanied by deteriorated CAN and increased course of diabetes [30]. The present study showed significant differences in pulse pressure among the NCAN, PCAN, and DCAN groups, with pulse pressure showing inverse correlations with HRV_DP and HRV 30 : 15.

Consequently, CAN was associated with left ventricular diastolic dysfunction, increased pulse pressure, and prolonged QTc. Further multivariable logistic analysis revealed CAN to be an independent risk factor for LVDD. It may be more meaningful to focus on cardiovascular disease outcomes in diabetic patients with CAN in future studies.

In conclusion, CAN was found to be an independent risk factor for LVDD in type 2 diabetes. The increase among the groups of NCAN, PCAN, and DCAN was correlated with deteriorated LVDD. Early diagnosis and treatment of CAN are advocated for preventing LVDD and related cardiovascular comorbidities in type 2 diabetes.

## Figures and Tables

**Table 1:** General characteristics of study population.

	NCAN	PCAN	DCAN	*P* value
*N*	36	161	118	—
Age (year)	54.2 ± 11.8	57.1 ± 11.2	60.4 ± 8.0	0.002^*∗∗*^
Duration of diabetes (year)	7.9 ± 6.3	10.2 ± 7.4	12.9 ± 7.6	0.000^*∗∗*^
Male sex (%)	72.2	61.3	57.6	0.290
BMI (kg/m^2^)	24.7 ± 3.5	25.2 ± 3.5	25.2 ± 4.0	0.768
Waistline (cm)	90.7 ± 7.1	91.6 ± 8.0	93.2 ± 9.9	0.215
FBG (mmol/L)	7.8 ± 2.1	8.0 ± 2.4	8.6 ± 2.4	0.065
HbA1c (%)	9.2 ± 1.7	8.9 ± 2.2	9.2 ± 2.1	0.440
Triglycerides (mmol/L)	2.01 ± 1.29	1.70 ± 1.02	1.82 ± 1.28	0.309
Total cholesterol (mmol/L)	4.29 ± 0.77	4.07 ± 1.18	4.49 ± 1.50	0.033^*∗*^
HDL cholesterol (mmol/L)	0.97 ± 0.31	1.05 ± 0.30	1.09 ± 0.33	0.133
LDL cholesterol (mmol/L)	2.28 ± 0.63	2.24 ± 0.84	2.52 ± 0.82	0.022^*∗*^
Pulse pressure (mmHg)	49.3 ± 9.0	50.6 ± 13.2	54.2 ± 12.4	0.027^*∗*^
Resting heart rate (bpm)	69.3 ± 8.5	69.1 ± 9.1	69.0 ± 10.6	0.991
QTc (ms)	407.6 ± 19.2	411.0 ± 16.9	415.5 ± 16.3	0.027^*∗*^
Retinopathy (%)	29.0	29.3	48.1	0.005^*∗∗*^
Nephropathy (%)	27.8	28.2	27.1	0.979
DSPN (%)	24.1	27.3	52.3	0.000^*∗∗*^
Current smoker (%)	44.4	26.4	28.8	0.097
Family history of diabetes (%)	22.2	36.8	52.5	0.002^*∗∗*^
History of hypertension (%)	38.9	58.3	55.1	0.957

Data are presented as means ± SD or *n* (%). BMI, body mass index; FBG, fasting blood glucose; HbA1c, glycosylated hemoglobin; HDL, high-density lipoprotein; LDL, low-density lipoprotein; QTc, corrected QT interval; DSPN, diabetic sensorimotor polyneuropathy. ^*∗*^*P* < 0.05; ^*∗∗*^*P* < 0.01.

**Table 2:** Assessment of left ventricular diastolic function by the stage of CAN.

	NCAN	PCAN	DCAN	*P* value
*N*	36	161	118	—
EF (%)	61.0 ± 2.3	60.2 ± 2.9	60.5 ± 1.9	0.228
*E* (cm/s)	0.74 ± 0.14	0.74 ± 0.18	0.71 ± 0.17	0.377
*A* (cm/s)	0.78 ± 0.20	0.77 ± 0.20	0.88 ± 0.16	0.000^*∗∗*^
*E*/*A*	1.01 ± 0.36	0.99 ± 0.32	0.83 ± 0.23	0.000^*∗∗*^
*e*′ (cm/s)	8.6 ± 2.4	8.3 ± 3.1	6.8 ± 1.7	0.000^*∗∗*^
*E*/*e*′	9.4 ± 2.8	9.8 ± 2.8	10.9 ± 2.7	0.006^*∗∗*^
LV mass (g)	145.9 ± 28.6	153.0 ± 31.6	167.5 ± 40.3	0.000^*∗∗*^
LVMI (g/m^2^)	83.8 ± 13.3	87.1 ± 16.1	95.8 ± 19.1	0.000^*∗∗*^
LVDD (%)	39.4	45.3	68.0	0.001^*∗∗*^

Data are presented as means ± SD or *n* (%). *E*, early diastolic filling velocity; *A*, late diastolic filling velocity; *e*′, early diastolic mitral annulus velocity; LVMI, left ventricular mass index; LVDD, left ventricular diastolic dysfunction. ^*∗∗*^*P* < 0.01.

**Table 3:** Correlation between CAN assessment parameters and echocardiography indicators for diastolic dysfunction.

	HRV_DP		HRV_Valsalva		HRV 30 : 15		BPV	
	*r*	*P*	*r*	*P*	*r*	*P*	*r*	*P*
*E*/*A*	0.156	0.009^*∗∗*^	0.136	0.117	0.309	0.000^*∗∗*^	0.072	0.202
*e*′	0.227	0.001^*∗∗*^	0.216	0.031^*∗*^	0.224	0.000^*∗∗*^	0.016	0.804
*E*/*e*′	−0.176	0.010^*∗*^	−0.205	0.041^*∗*^	−0.211	0.001^*∗∗*^	0.095	0.143
LV mass	−0.203	0.001^*∗∗*^	−0.118	0.172	−0.131	0.020^*∗*^	−0.081	0.150
LVMI	−0.158	0.009^*∗∗*^	−0.115	0.183	−0.211	0.000^*∗∗*^	−0.086	0.134

*E*, early diastolic filling velocity; *A*, late diastolic filling velocity; *e*′, early diastolic mitral annulus velocity; LVMI, left ventricular mass index; HRV_DP, heart rate variation during deep breathing; HRV_Valsalva, heart rate variation during Valsalva maneuver; HRV 30 : 15, heart rate variation during the lying-to-standing test; BPV, blood pressure variability in response to standing up. ^*∗*^*P* < 0.05; ^*∗∗*^*P* < 0.01.

**Table 4:** Multivariate analysis of the correlation between selected variables and LVDD.

Variables	OR	95% CI	*P* value
Age (year)	1.052	1.022–1.083	0.001^*∗∗*^
Gender (male/female)	1.483	0.451–4.790	0.513
Duration of diabetes (year)	1.009	0.971–1.049	0.644
Current smoker (yes/no)	1.449	0.797–2.635	0.224
LDL (mmol/L)	0.905	0.683–1.119	0.498
HbA1c (%)	0.894	0.732–1.069	0.222
Pulse pressure (mmHg)	0.993	0.938–1.042	0.778
QTc (ms)	0.981	0.957–1.016	0.379
CAN stage	1.628	1.131–2.344	0.009^*∗∗*^

LVDD, left ventricular diastolic dysfunction; LDL, low-density lipoprotein; HbA1c, glycosylated hemoglobin; QTc, corrected QT interval; CAN, cardiovascular autonomic neuropathy. ^*∗∗*^*P* < 0.01.

## References

[1] Spallone V., Ziegler D., Freeman R. (2011). Cardiovascular autonomic neuropathy in diabetes: clinical impact, assessment, diagnosis, and management. *Diabetes Metabolism Research and Reviews*.

[2] Ziegler D. (1994). Diabetic cardiovascular autonomic neuropathy: Prognosis, diagnosis and treatment. *Diabetes Metabolism Reviews*.

[3] Mogensen U. M., Jensen T., Køber L. (2012). Cardiovascular autonomic neuropathy and subclinical cardiovascular disease in normoalbuminuric type 1 diabetic patients. *Diabetes*.

[4] Ziegler D., Gries A. F., Muhlen H. (1993). Prevalence and clinical correlates of cardiovascular autonomic and peripheral diabetic neuropathy in patients attending diabetes centers. The Diacan Multicenter Study Group. *Diabete Metab*.

[5] Pop-Busui R., Low P. A., Waberski B. H. (2009). Effects of prior intensive insulin therapy on cardiac autonomic nervous system function in type 1 diabetes mellitus: the diabetes control and complications trial/epidemiology of diabetes interventions and complications study (DCCT/EDIC). *Circulation*.

[6] Vinik A. I., Ziegler D. (2007). Diabetic cardiovascular autonomic neuropathy. *Circulation*.

[7] Maser R. E., Mitchell B. D., Vinik A. I., Freeman R. (2003). The association between cardiovascular autonomic neuropathy and mortality in individuals with diabetes a meta-analysis. *Diabetes Care*.

[8] Pop-Busui R., Evans G. W., Gerstein H. C. (2010). Effects of cardiac autonomic dysfunction on mortality risk in the Action to Control Cardiovascular Risk in Diabetes (ACCORD) trial. *Diabetes Care*.

[9] Soedamah-Muthu S. S., Chaturvedi N., Witte D. R., Stevens L. K., Porta M., Fuller J. H. (2008). Relationship between risk factors and mortality in type 1 diabetic patients in europe: The eurodiab prospective complications study (PCS). *Diabetes Care*.

[10] Vinik A. I., Freeman R., Erbas T. (2003). Diabetic autonomic neuropathy. *Seminars in Neurology*.

[11] Rossing P., Breum L., Major-Pedersen A. (2001). Prolonged QTc interval predicts mortality in patients with type 1 diabetes mellitus. *Diabetic Medicine*.

[12] Bell D. S. H. (2003). Heart failure: the frequent, forgotten, and often fatal complication of diabetes. *Diabetes Care*.

[13] Bhatia R. S., Tu J. V., Lee D. S. (2006). Outcome of heart failure with preserved ejection fraction in a population-based study. *The New England Journal of Medicine*.

[14] Poanta L., Porojan M., Dumitrascu D. L. (2011). Heart rate variability and diastolic dysfunction in patients with type 2 diabetes mellitus. *Acta Diabetologica*.

[15] Pop-Busui R., Cleary P. A., Braffett B. H. (2013). Association between cardiovascular autonomic neuropathy and left ventricular dysfunction: DCCT/EDIC study (diabetes control and complications trial/epidemiology of diabetes interventions and complications). *Journal of the American College of Cardiology*.

[16] Sacre J. W., Franjic B., Jellis C. L., Jenkins C., Coombes J. S., Marwick T. H. (2010). Association of cardiac autonomic neuropathy with subclinical myocardial dysfunction in type 2 diabetes. *JACC: Cardiovascular Imaging*.

[17] Novak P. (2011). Quantitative autonomic testing. *Journal of Visualized Experiments*.

[18] Mythri S., Rajeev H. (2015). Left ventricular diastolic dysfunction (LVDD) & cardiovascular autonomic neuropathy (CAN) in type 2 diabetes mellitus (DM): a cross-sectional clinical study. *Journal of Clinical and Diagnostic Research*.

[19] Mogelvang R., Sogaard P., Pedersen S. A. (2009). Cardiac dysfunction assessed by echocardiographic tissue doppler imaging is an independent predictor of mortality in the general population. *Circulation*.

[20] Nagueh S. F., Appleton C. P., Gillebert T. C. (2009). Recommendations for the evaluation of left ventricular diastolic function by echocardiography. *European Journal of Echocardiography*.

[21] Wan S. H., Vogel M. W., Chen H. H. (2014). Pre-clinical diastolic dysfunction. *Journal of the American College of Cardiology*.

[22] Poulsen M. K., Henriksen J. E., Dahl J. (2010). Left ventricular diastolic function in type 2 diabetes mellitus prevalence and association with myocardial and vascular disease. *Circulation Cardiovascular Imaging*.

[23] Imam M. H., Karmakar C. K., Jelinek H. F., Palaniswami M., Khandoker A. H. (2015). Analyzing systolic-diastolic interval interaction characteristics in diabetic cardiac autonomic neuropathy progression. *IEEE Journal of Translational Engineering in Health and Medicine*.

[24] Habek J. C., Lakusic N., Kruzliak P., Sikic J., Mahovic D., Vrbanic L. (2014). Left ventricular diastolic function in diabetes mellitus type 2 patients: correlation with heart rate and its variability. *Acta Diabetologica*.

[25] Dinh W., Futh R., Lankisch M. (2011). Cardiovascular autonomic neuropathy contributes to left ventricular diastolic dysfunction in subjects with type 2 diabetes and impaired glucose tolerance undergoing coronary angiography. *Diabetic Medicine*.

[26] Pappachan J. M., Sebastian J., Bino B. C. (2008). Cardiac autonomic neuropathy in diabetes mellitus: prevalence, risk factors and utility of corrected QT interval in the ECG for its diagnosis. *Postgraduate Medical Journal*.

[27] Khandoker A. H., Imam M. H., Couderc J.-P., Palaniswami M., Jelinek H. F. (2012). QT variability index changes with severity of cardiovascular autonomic neuropathy. *IEEE Transactions on Information Technology in Biomedicine*.

[28] de Bruyne M. C., Hoes A. W., Kors J. A. (1999). Prolonged QT interval predicts cardiac and all-cause mortality in the elderly. *European Heart Journal*.

[29] Schram M. T., Kostense P. J., Van Dijk R. A. (2002). Diabetes, pulse pressure and cardiovascular mortality: the hoorn study. *Journal of Hypertension*.

[30] Philips J. C., Marchand M., Scheen A. J. (2009). Pulse pressure and cardiovascular autonomic neuropathy according to duration of type 1 diabetes. *Diabetes Metabolism Research and Reviews*.

